# The Ronne Ice Shelf survived the last interglacial

**DOI:** 10.1038/s41586-024-08394-w

**Published:** 2025-01-29

**Authors:** Eric W. Wolff, Robert Mulvaney, Mackenzie M. Grieman, Helene M. Hoffmann, Jack Humby, Christoph Nehrbass-Ahles, Rachael H. Rhodes, Isobel F. Rowell, Louise C. Sime, Hubertus Fischer, Thomas F. Stocker, Amaelle Landais, Frédéric Parrenin, Eric J. Steig, Marina Dütsch, Nicholas R. Golledge

**Affiliations:** 1 Department of Earth Sciences, University of Cambridge, Cambridge, UK; 2 British Antarctic Survey, Cambridge, UK; 3 Geo- and Environmental Center, University of Tübingen, Tübingen, Germany; 4 National Physical Laboratory, Teddington, UK; 5 Climate and Environmental Physics, Physics Institute, and Oeschger Centre for Climate Change, University of Bern, Bern, Switzerland; 6 Laboratoire des Sciences du Climat et de l’Environnement, LSCE/IPSL, CEA-CNRS-UVSQ, Université Paris-Saclay, Gif-sur-Yvette, France; 7 Université Grenoble Alpes, CNRS, INRAE, IRD, Grenoble INP, IGE, Grenoble, France; 8 Department of Earth and Space Sciences and Department of Atmospheric Sciences, University of Washington, Seattle, WA USA; 9 Department of Meteorology and Geophysics, University of Vienna, Vienna, Austria; 10 Antarctic Research Centre, Victoria University of Wellington, Wellington, New Zealand

**Keywords:** Palaeoclimate, Cryospheric science

## Abstract

The fate of the West Antarctic Ice Sheet (WAIS)^1^ is the largest cause of uncertainty in long-term sea-level projections. In the last interglacial (LIG) around 125,000 years ago, data suggest that sea level was several metres higher than today^2–4^, and required a significant contribution from Antarctic ice loss, with WAIS usually implicated. Antarctica and the Southern Ocean were warmer than today^5–8^, by amounts comparable to those expected by 2100 under moderate to high future warming scenarios. However, direct evidence about the size of WAIS in the LIG is sparse. Here we use sea salt data from an ice core from Skytrain Ice Rise, adjacent to WAIS, to show that, during most of the LIG, the Ronne Ice Shelf was still in place, and close to its current extent. Water isotope data are consistent with a retreat of WAIS^9^, but seem inconsistent with more dramatic model realizations^10^ in which both WAIS and the large Antarctic ice shelves were lost. This new constraint calls for a reappraisal of other elements of the LIG sea-level budget. It also weakens the observational basis that motivated model simulations projecting the highest end of projections for future rates of sea-level rise to 2300 and beyond.

## Abstract (web summary)

Sea salt data from an ice core record show that Antarctica’s Ronne Ice Shelf survived the last interglacial, the last period of enhanced and sustained global warmth about 125,000 years ago.

## Main

The West Antarctic Ice Sheet (WAIS) is considered particularly vulnerable to retreat due to climate change^11^, primarily because it is mainly marine-based, sitting on a bed below sea level. The reverse bed slope (deepening inland) beneath the margins of much of WAIS makes it potentially subject to irreversible retreat due to marine ice sheet instability (MISI)^12–14^. The range of projections of future sea-level contributions from Antarctica for a given climate scenario is large^11^. A number of ice-sheet modelling studies project loss of much of WAIS under the highest warming scenarios (for example, representative concentration pathway RCP 8.5)^15,16^, but this loss generally occurs over several centuries. By the year 2200 ce, most of these models predict only a few tens of centimetres of sea-level rise from Antarctica (particularly WAIS), even under RCP 8.5 (refs. ^15–17^). However, one model, which applied a large ocean warming, and which includes the extra proposed mechanisms of hydrofracturing and marine ice cliff instability (MICI)^18^, predicts much faster and greater loss of ice from WAIS and other parts of Antarctica, reaching the equivalent of 5 m of sea-level rise by 2200^10,19^.

The model configuration and ocean forcing of these studies^10,19^ were partly motivated by consideration of possible ice loss during the last interglacial (LIG, about 130–115 thousand years ago (ka)). Both Greenland^20^ and Antarctica^6,7^ were warmer during the LIG than at present. Synthesis of relative sea-level data, derived mainly from corals and interpreted using glacial isostatic modelling, has suggested that global mean sea level during the LIG may have been 6–9 m higher than today^2,11^. After taking account of a contribution of up to 1 m from thermosteric expansion of seawater^21^ and mountain glaciers, a large contribution from the Greenland and Antarctic ice sheets is required to reach this higher sea level. Most studies estimate at most a 2-m LIG sea-level input from the Greenland Ice Sheet^22–24^, which suggests a substantial Antarctic sea-level contribution. One study estimates^25^ a greater contribution from Greenland of 5.1 m, but because this study constrains peak input to late in the LIG (about 121 ka), it still requires a substantial Antarctic input at 125 ka. More recent studies estimating a LIG global eustatic sea-level rise of 1.2–5.3 m above present^3^ or even 0.4–2.7 m above present^4^ reduce the requirement for Antarctic ice loss. Nonetheless, most studies have inferred from the sea-level and Greenland data that the size of the Antarctic Ice Sheet (and probably WAIS) must have been substantially reduced in the LIG^26^.

Only a few ice-sheet modelling studies have tried to predict the state of the Antarctic Ice Sheet during the LIG, and their estimates of ice loss from Antarctica range from zero in the absence of significant ocean warming^27^, up to 7.5 m of sea-level equivalent^10^. The study that included the MICI mechanism^10^ produced a LIG Antarctic ice loss of greater than 6 m sea-level equivalent, including complete loss of the Ronne–Filchner and Ross ice shelves. Other work^9,28^ suggested a more modest loss of ice in the WAIS region, with up to 3 m (ref. ^28^) or 4 m (ref. ^9^) of sea-level equivalent compared to today. In these studies, loss was focussed in the Amundsen Sea region, and the Ronne Ice Shelf was retained at close to its present configuration.

Direct proximal evidence of the state of WAIS during the LIG has so far been missing. However, a recent paper^29^ studying the genomics of an octopus species around the Antarctic continent suggests that seaways may have existed between the Ronne, Amundsen and Ross embayments, with a timing which is inferred to represent the LIG. This would imply a substantial loss of ice in the LIG.

**Fig. 1:**
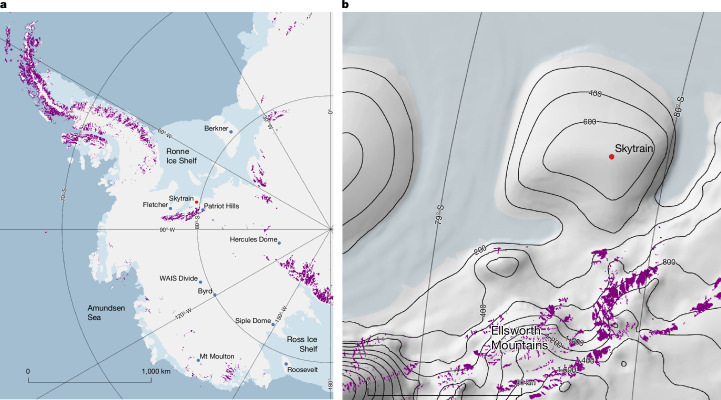
The location of the SIR drilling site. SIR is shown as a red dot and other existing and proposed ice core sites around WAIS as blue dots. The purple colour shows exposed rock outcrops. **a**, Overview of West Antarctica. **b**, Detail of the SIR region. Contours are from the Cryosat2 elevation model^42^. Maps in **a** and **b** were generated using QGIS with the Quantarctica mapping environment^43^, under a Creative Commons licence CC BY 4.0.

The only published LIG ice core record from WAIS is a horizontal ice trench record from Mount Moulton (Fig. 1)^30^. An early interpretation of the differences in the LIG water isotope data between Mount Moulton and East Antarctic sites, using isotope-enabled climate model output, led to a tentative qualitative interpretation that WAIS did collapse in the LIG^31^. However, a more comprehensive recent modelling study^32^ did not support this conclusion, and instead suggested that the Mount Moulton isotope record is rather insensitive to WAIS collapse. A horizontal ice trench record from blue ice in the Patriot Hills (Fig. 1)^33^ suggested a LIG hiatus in ice deposition or preservation that was initially interpreted as supporting substantial LIG mass loss in the Weddell Sea embayment. However, subsequent work has suggested a different interpretation involving changes in ice flow induced by bedrock uplift and more modest local thinning^9^.

Here we present data from the Skytrain Ice Rise (SIR) ice core, which provides proxy-based estimates of ice shelf extent, local ice elevation and, in combination with data from other cores and studies, constraints on the extent of WAIS ice loss in the LIG.

## Skytrain Ice Rise

SIR is an ice cap, independent from the main body of WAIS, that sits at the landward margin of the Ronne Ice Shelf adjacent to WAIS (Fig. 1). SIR is expected to have remained an independent ice cap throughout the last glacial cycle because it is separated from the main ice sheet by the high barrier of the Ellsworth Mountains and it has bedrock sitting above sea level surrounded radially almost entirely by bathymetry greater than 1,000 m.

An ice core was drilled to bedrock at SIR (651 m depth)^34^, and was subsequently dated, giving an age model tied to those of other Antarctic ice cores^35^. Although there is evidence of flow disturbance in the lowest 50 m of the ice (beyond 108 ka), ice from 117–126 ka (617–627 m) in the LIG is present and is in stratigraphic order^35^ (Methods). Unfortunately, ice from the very warmest part of the LIG between 127 and 129 ka is missing from our record, even though older ice is present below the LIG section^35^.

## SIR sea salt record

Sodium (Na) is used as a sea salt indicator in the ice core record. Sea salt is transported and deposited to ice sheets from the saline surfaces of sea ice and the open ocean. Its concentration in ice cores is therefore influenced by the pattern and strength of atmospheric transport and by sea ice extent^36^. To reach SIR, sea salt must travel from the ice front across the non-marine Ronne Ice Shelf, whose front is now 680 km from the drill site. As a result, a first-order control on sea salt concentration at SIR is distance from the source (the ice shelf edge) to SIR. A spatial survey along the Ronne Ice Shelf^37^ shows a rapid decrease in sea salt concentration with distance inland from the ice shelf front, demonstrating the predominant influence of the Weddell Sea on sea salt deposition across the ice shelf. If the ice shelf disintegrated, bringing salty surfaces closer to SIR, we would expect much higher than present Na concentration.

**Fig. 2:**
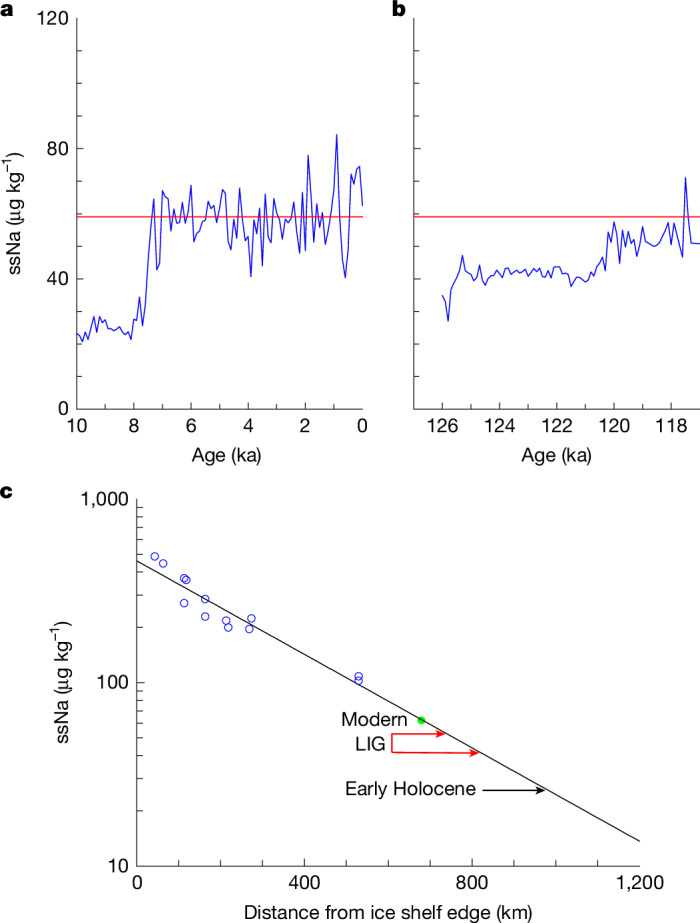
Sea salt sodium (ssNa) concentration at SIR comparing the Holocene and LIG. **a**, Holocene (10–0 ka, 100 year averages). **b**, LIG (126–117 ka, 100 year averages). The red line in **a** and **b** is the average value for 7–0 ka. **c**, Data from a published spatial survey (blue circles) of sea salt concentrations on the Ronne Ice Shelf^37^. The solid black line is the best fit^38^ and the green circle is the present concentration at SIR (top 25 m of core) which fits closely onto the line. The vertical position of the black arrow is the average ssNa concentrations from 10–8 ka, when the Ronne Ice Shelf was extended. The red arrows labelled LIG indicate average ssNa values from 125–121 ka (lower arrow) and 120–118 ka (upper arrow).

**Extended Data Fig. 1:**
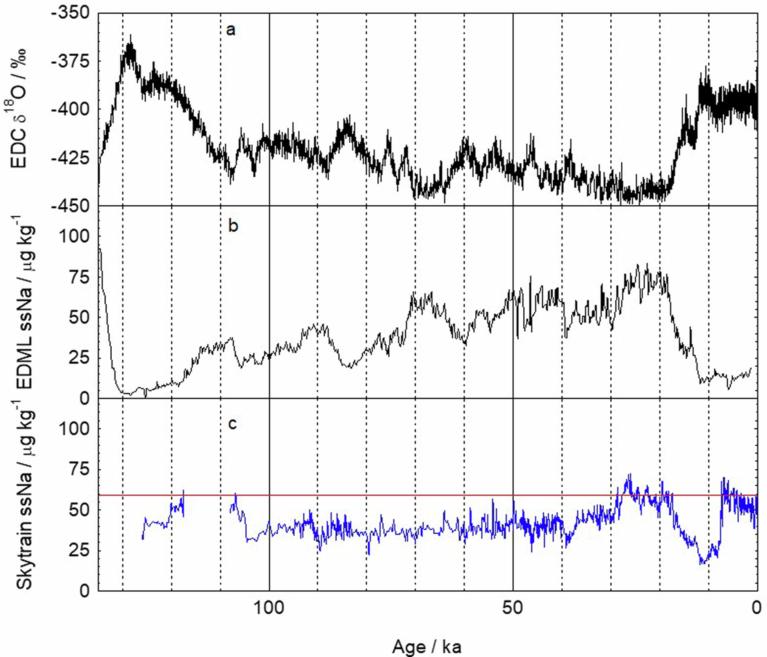
ssNa data over the last glacial cycle, showing the interglacials in context. (a) Deuterium record of climate at Dome C^6^ to provide context for the progression of climate across Antarctica. (b) ssNa at EDML^61^ for comparison with SIR(c) ssNa at SIR (100 year means, dated sections only); a datapoint at 481.0 m where there is a visible ash layer is not plotted. The red line shows the average concentration of ssNa at SIR in the period from 7-5 ka.

In fact, we find that Na concentration across the warm plateau of the LIG from 126 to 117 ka was lower than today, reaching late Holocene concentrations only late in the LIG, at 120 ka (Fig. 2, Extended Data Table 1 and Extended Data Fig. 1). Although we cannot rule out some effects from changing transport and/or sea ice, this low Na concentration suggests that the ice shelf could actually have been extended beyond its current position at 126 ka, as it was in the early Holocene^37^. It then retreated to near its current position at about 120 ka. This would be consistent with what occurred in the Holocene, when the ice shelf edge was extended beyond its current position in the early Holocene, and retreated to its current position only at 7.3 ka (ref. ^38^). There is no sign of the concentrations of around 400 µg kg^−1^ that the spatial survey shown in Fig. 2 suggests would be expected if the ice shelf disintegrated completely.

## SIR water isotope record

Like sea salt, the water isotope signal at SIR in the LIG results from a combination of environmental changes. The differences between LIG and Holocene values will be a response to three components: (1) change in climate (temperature and sea ice); (2) local elevation change at SIR and (3) change in large-scale circulation resulting from changes in the morphology of Antarctica, here potentially the loss of WAIS. If components (1) and (2) can be estimated then we can adjust the measured data to provide component (3), which is diagnostic of large-scale changes in the WAIS. Recent modelling work^32^ found that if WAIS was almost completely lost in the LIG^39^, δ^18^O at SIR would increase by about 4‰ compared to present, after accounting for a local change in elevation and assuming a modern climate.

**Fig. 3:**
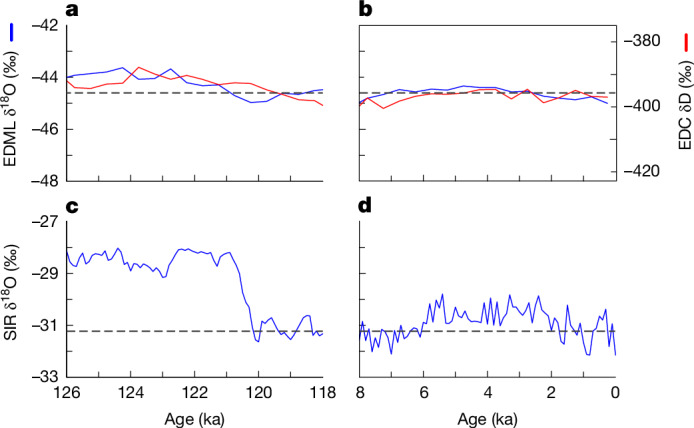
δ^18^O for the SIR ice and East Antarctic cores in the LIG and Holocene. **a**, Reference data for the LIG for EPICA Dome C (EDC)^6^ (red, δD) and EPICA Dronning Maud Land (EDML)^44^ (blue, δ^18^O), scaled 8:1, with the horizontal line being an indicative level for the late Holocene. **b**, As **a** but for the last 8 kyr. **c**, SIR data for the LIG. **d**, SIR data for the last 8 kyr. The dashed line in **c** and **d** is the average of 2–0 ka. All three records are synchronized to the AICC2012 age model. The *y*-scaling is the same for all three cores and both time periods.

**Extended Data Fig. 2:**
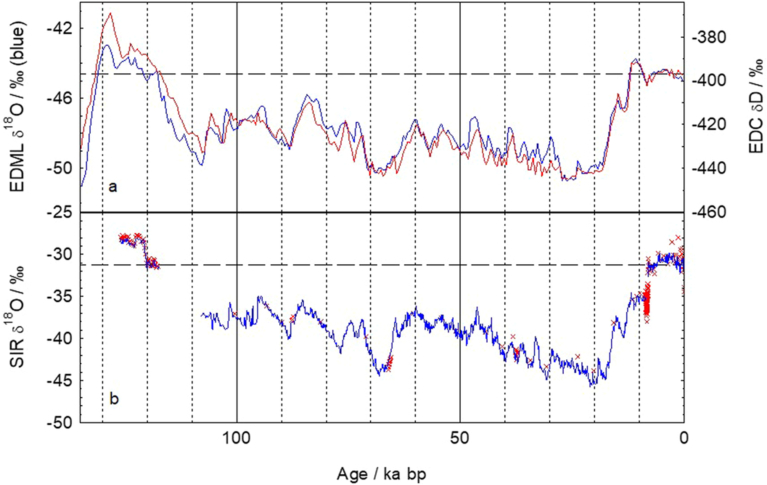
δ^18^O for the the SIR ice and East Antarctic cores over the last glacial cycle. (a) Reference data for EDC^6^ (red, δD) and EDML^44^ (blue, δ^18^O), scaled 8:1, with the horizontal line being an indicative level for the late Holocene. (b) SIR core data, shown only for the sections with reliable dates. The red crosses are discrete measurements, while the blue line is those made from the CFA meltstream. The dashed line is the average of 2-0 ka. All 3 records are synchronised to the AICC2012 age model. Note that the y-scaling is different in the two panels.

The expected Antarctic spatial pattern of δ^18^O due to LIG climate (component (1)) can be estimated from model simulations, in which orbital and greenhouse gas values are changed from pre-industrial values to those of the LIG and all other parameters are kept constant. Such model simulations produce only a small net change in isotopic values^40^ but with spatial variability of about 1‰ across the continent. This is consistent with measured δ^18^O values for 120–126 ka at five East Antarctic reference sites, which show an average increase of +0.7‰ (range 0.3–1.3‰) relative to those of the last 7.5 kyr (Fig. 3, Extended Data Fig. 2 and Extended Data Table 2), although this may also contain an element due to elevation change^32^. By contrast, the δ^18^O value at SIR between 126 and 120 ka is much larger: +2.5‰ above the late Holocene (2–0 ka) value (or +2.2‰ above that of the last 7.5 ka). This suggests that, after removing the signal arising from continent-wide changes in climate, the net isotopic signal at SIR due to changes in site elevation and large-scale changes in circulation is probably between about 1 and 2‰.

**Extended Data Fig. 3:**
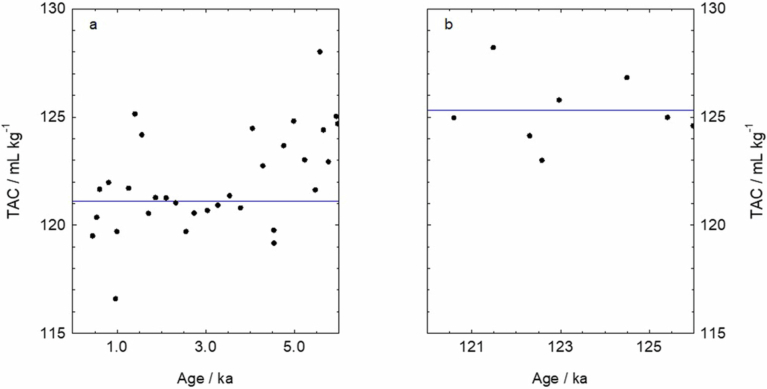
Total air content data (without cut bubble correction) from SIR. (a) Data for 0-6 ka, (b) for 120-126 ka. Only data with weight ranging between 40 and 80 g are shown, to limit the deviations that would be caused by cut bubbles within each depth span. The blue lines are the SIR averages (2-0.45 ka, 126-120 ka) used in the text.

To constrain component (2) (site elevation) we consider total air content (TAC) of the ice. SIR TAC (without cut-bubble correction; Methods) for the LIG (average of 126–120 ka) was 125.3 ± 0.6 ml kg^−1^ (mean ± s.e.m.), which is higher than the late Holocene (2–0.45 ka) value of 121.1 ± 0.7 ml kg^−1^ (Extended Data Fig. 3). Adjusting for the different cut-bubble correction in the two time periods caused by the reduction in bubble size under pressure deeper in the ice sheet (Methods) and accounting for the temperature change estimated from the measured change in δ^18^O (Methods), we estimate that the elevation at Skytrain in the LIG was either 150 ± 90 m lower (if the pore volume *V*_c_ was invariant between the two periods) or 60 ± 80 m lower (if pore volume varied with temperature). With an estimated δ^18^O-elevation gradient of 0.8 ± 0.2‰ per 100 m (Methods), local changes in altitude could account for up to 2‰ of the 2.5‰ change in δ^18^O at SIR between the LIG and the Holocene.

Combining these estimates of components (1) and (2) suggests that component (3), from changes in circulation resulting from large-scale changes to the topography of the WAIS, is between 0 and 2‰. Although this value is uncertain, it is at most about half that predicted by the high-resolution isotope model^32^ for the case of a near-complete WAIS collapse. Nonetheless, the water isotope values at SIR in the LIG are significantly higher than those in the Holocene, which certainly indicates loss of ice, either at SIR itself (elevation effect) or across WAIS more generally, and probably both.

## WAIS, Ronne Ice Shelf and the LIG

**Extended Data Fig. 4:**
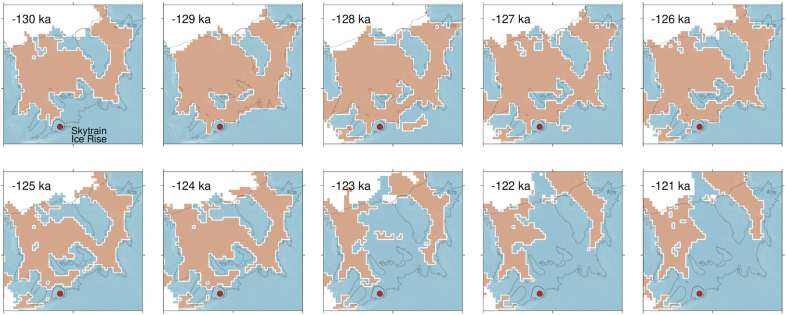
A time series of modelled ice sheet and ice shelf extent in the Weddell Sea sector of Antarctica. This is the time series for the model run highlighted in Fig. 4, which shows the 127 ka time slice in more detail. Model output maps were plotted using Generic Mapping Tools v.6.

Our Na results indicate that the Ronne Ice Shelf was intact between 126 and 120 ka, and may have been more extensive than today in the early part of that period. Although we have no data for the period of maximum Antarctic warmth between 130 and 126 ka, it is unlikely that the ice shelf disappeared and then regrew under the warm, low sea ice, conditions of this period. Probabilistic sea-level reconstructions suggest that the LIG sea-level maximum occurred at or after 126 ka (ref. ^41^), arguing against substantial ice growth before then. Furthermore, model simulations show that, despite increasing mass loss from the continent during this interval, the extent of the Ronne Ice Shelf remained largely unchanged (Extended Data Fig. 4). We therefore conclude that the Ronne Ice Shelf survived the LIG, with a minimum extent similar to or greater than that of the present.

**Fig. 4:**
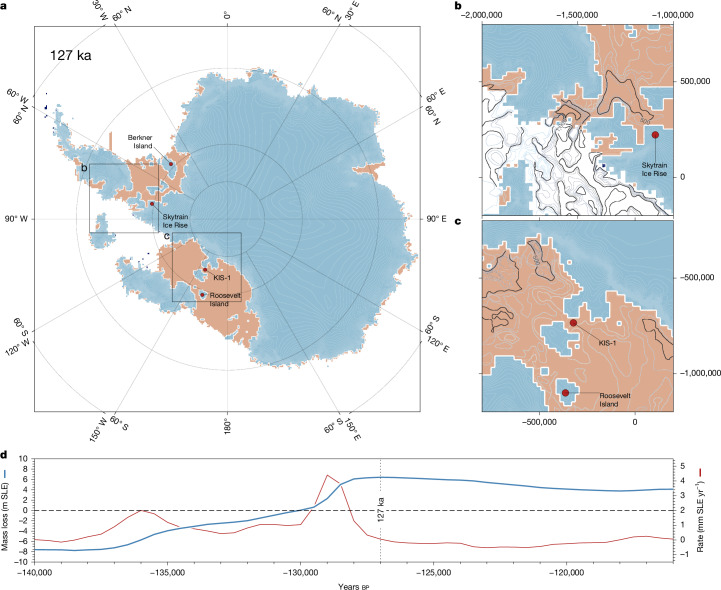
A model reconstruction of the Antarctic Ice Sheet at the LIG. The model is consistent with both our data showing the presence of ice shelves (absence of exposed ocean or sea ice) and data indicating the existence of seaways. **a**–**c**, Antarctica (**a**); close-up of region around SIR (**b**); close-up of region in Ross Ice Shelf (**c**). In **a**–**c**, blue is grounded ice, pink is ice shelf. **b** and **c** show water column thickness contours, with units of metres, for the ocean beneath the ice shelves. **d**, Time series of modelled magnitude (blue) and rate (red) of sea-level-equivalent mass loss for the shown scenario. Model output maps were plotted using Generic Mapping Tools v.6.

A recent study suggested that LIG seaways accounted for observed gene transfer between the Weddell, Amundsen and Ross Sea sectors of Antarctica in a species of deep-living octopus^29^. These two sets of results are not mutually exclusive, but can be reconciled with an Antarctic configuration in which ice in the Amundsen Sea sector was significantly reduced, but the ice shelves, fed by ice streams in the Ross and Ronne catchments, were intact. This also implies that the inferred seaways existed at depth but were covered by ice shelf and did not represent a source of open water, sea ice or sea salt aerosol. Figure 4 illustrates a possible ice sheet/shelf configuration that reconciles our new data from SIR with that from the octopus study^29^. The simulation shown is derived from a previously published ensemble^9^. In this scenario, loss of WAIS occurs as a consequence of ocean thermal driving in the Amundsen Sea embayment that triggers marine ice sheet instability. However, CCSM3-simulated cooler-than-present ocean temperatures in the Ross and Weddell Seas facilitate ice shelf preservation in these larger two embayments. The result is an ice geometry in which Na flux to SIR from the Weddell Sea is low throughout the interglacial (Methods).

The net water isotopic signal at SIR between 126 and 120 ka (compared to the Holocene) is less (perhaps half) than that predicted for complete loss of WAIS^32^. In the absence of isotope-enabled climate model simulations at high resolution involving a partial collapse, or of robust results from other models with enough resolution to diagnose the response at SIR, we conclude that our results are consistent with a partial (but not full) collapse of WAIS, focussed on the Amundsen Sea sector. We again can draw no direct conclusions for the period 130–126 ka, but we suggest that regrowth of WAIS under such warm conditions is unlikely to have occurred.

Our findings are compatible with ice-sheet model simulations in which retreat occurred mainly in the Amundsen Sea sector^9^, but they are not consistent with simulations in which ocean warming and MICI led to great loss of ice in the Weddell (and Ross) Sea sectors^10^. This has some important consequences. First, if the main centre of ice loss was the Amundsen Sea sector, with continued ice shelf buttressing of ice flowing into the Ronne and Ross ice shelves, then the magnitude and rate of LIG sea-level rise above present as a result of Antarctic ice loss are probably well below those proposed in ref. ^10^. Although estimates of LIG sea-level contributions from Antarctica in simulations that retain the large ice shelves vary across ensembles of model realizations (for example, Fig. 4), values of 4 m (ref. ^9^) are cited, and lower values still could be expected if not all the Amundsen Sea sector ice was lost, as our small isotopic change might suggest. This is sufficient to explain more modest recent estimates of LIG sea-level rise^3,4^, but cannot be reconciled with the larger values used in many reviews and earlier studies^1,2,26^ unless Greenland contributed earlier than expected or there was a significant contribution from East Antarctica. A second conclusion is that our finding that the Ronne Ice Shelf survived the LIG weakens the observational basis to support projections of the most extreme WAIS loss by 2200, as suggested by simulations involving significant LIG sub-ice shelf ocean warming and the MICI mechanism^10,19^. Finally, our results refine the recent evidence from octopus DNA^29^ suggesting that WAIS was significantly reduced in size during a part of the LIG which was only modestly warmer than today. They do, however, pose a challenge to understand how long a period of seaway opening is sufficient to lead to the genomic findings, and whether there are mechanisms that can explain how the genetic material is transported under several hundred kilometres of covered ice shelf. Further ice cores reaching the LIG, such as that planned for Hercules Dome, along with high-resolution modelling of a range of possible WAIS configurations, will be crucial to advance our use of the LIG to constrain future Antarctic ice loss.

## Methods

The ice core at SIR was drilled to bedrock at 651 m in the field season 2018–2019^34^, and analysed using continuous flow analysis (CFA) as well as discrete sampling between 2019 and 2021^45^.

### Dating

**Extended Data Fig. 5:**
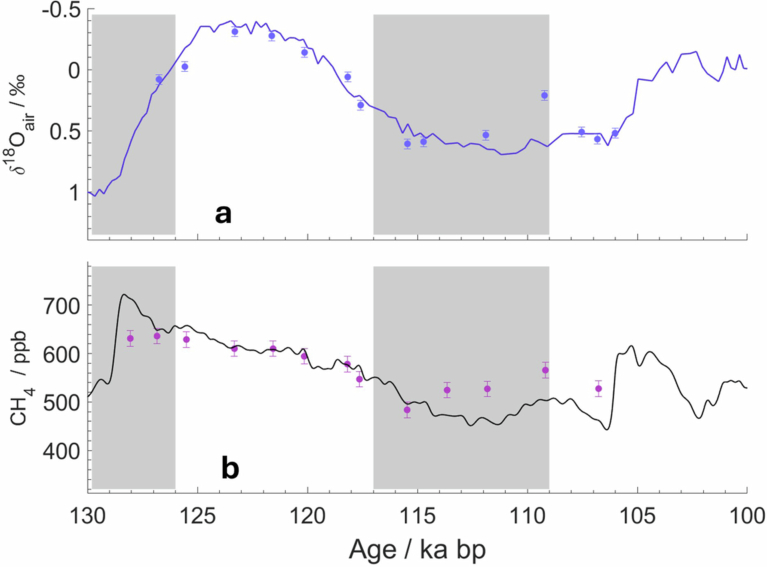
SIR CH_4_ and δ^18^O_atm_ between 100 and 130 ka emphasizing the robust nature of the dating for the LIG section used in this paper. (a) δ^18^O_atm_; discrete measurements from SIR shown as symbols, while the solid line is reference data.(b) Same as (a) but for CH_4_. Sections with unreliable ages (605-617 m and >627 m) are greyed out. The error bars are the combined uncertainty (at 1 sigma) of the Skytrain and reference data. Figure reproduced from ref. ^35^, Copernicus Publications, under a Creative Commons licence CC BY 4.0.

Beyond the top 2,000 years^46^, the ST22 age scale used here was created using the Paleochrono inverse model and a range of tie points that fix the age scale to AICC2012 ages^35^. Tie points came from ice chemistry and ^10^Be in the ice and from CH_4_ and δ^18^O_atm_ in the air. It was established that there is flow disturbance below 605 m (108 ka), and that ice from the early peak of the LIG (126–130 ka) and the penultimate glacial maximum is missing. Nonetheless the combined use of CH_4_ and δ^18^O_atm_ across the main part of the LIG provides clear evidence that the section from 117 to 126 ka is present and in the correct sequence. Paired CH_4_–δ^18^O_atm_ values are found that have not occurred anywhere else in the last 200 ka other than in the Holocene and in the LIG around 122 ka (Extended Data Fig. 5). The estimated uncertainty in age during the Holocene reference period is about 100 years. The quoted uncertainty for the LIG is less than 300 years, although it is difficult, given the flow disturbance above and below this section, to be sure that there are not nonlinear age–depth relationships between the CH_4_/δ^18^O_atm_ measurements, which are 1–2 kyr apart.

We do not know exactly why ice from about 127–140 ka, below the dated LIG section, is missing. Detailed examination of CH_4_/δ^18^O_atm_ values^35^ in four data points in the 5 m of ice immediately below the LIG section match reference data with ages 140–180 ka. Looking further up the core, we find no possible matches younger than this until at least 60 ka in three cases. Given that 60 ka ice is 80 m higher up the core in a region with flat radar horizons and no evidence of disturbance, we feel confident in saying that ice of age 140–180 ka is present below our LIG section^35^, which suggests that ice remained present at SIR throughout the penultimate glacial maximum and LIG, as predicted in all ice modelling studies of the LIG that we are aware of. As discussed in more detail elsewhere^35^, we conclude that flow disturbance caused by interaction between ice of different rheologies is responsible for the missing ice.

### Analyses

Na and Ca were measured using ICP-MS and CFA^45^. The ssNa was calculated by correcting for the terrestrial component,

1 $${\rm{ssNa}}=\frac{{R}_{{\rm{t}}}[{\rm{Na}}]-[{\rm{Ca}}]}{{R}_{{\rm{t}}}-{R}_{{\rm{m}}}}$$

where *R*_t_ and *R*_m_ are the weight ratio of Ca to Na in average crust and in seawater (1.78 and 0.038), respectively^38,47^. The average correction for both the Holocene (0–10 ka) and the LIG (120–126 ka) is only 3%. The uncertainty in concentration between the different periods shown is determined by the uncertainty in calibration, which was estimated at 1.6% (ref. ^45^).

Stable water isotopes (δ^18^O and δD) were measured continuously on the CFA meltstream using a Picarro L2130-I cavity ring down spectrometer^38^. The continuous measurements were compared with those made on discrete samples using a similar instrument but that could be more carefully and regularly calibrated. The discrete samples were taken approximately once every 10 m for the Holocene, whereas, for the LIG, discrete samples were collected throughout at 10 or 20-cm intervals.

Only the discrete samples were used to calculate deuterium excess (dxs = δD − 8δ^18^O), which is particularly sensitive to small uncertainties in the calibration of the two isotopes. The 24 deuterium excess values for the last 2 kyr have a mean of 6.1‰ with an s.d. of 1.1‰ (s.e.m. 0.22‰); for 126–120 ka, 33 discrete values have a mean of 4.9‰, with an s.d. of 0.65‰ (s.e.m. 0.11‰).

**Extended Data Fig. 6:**
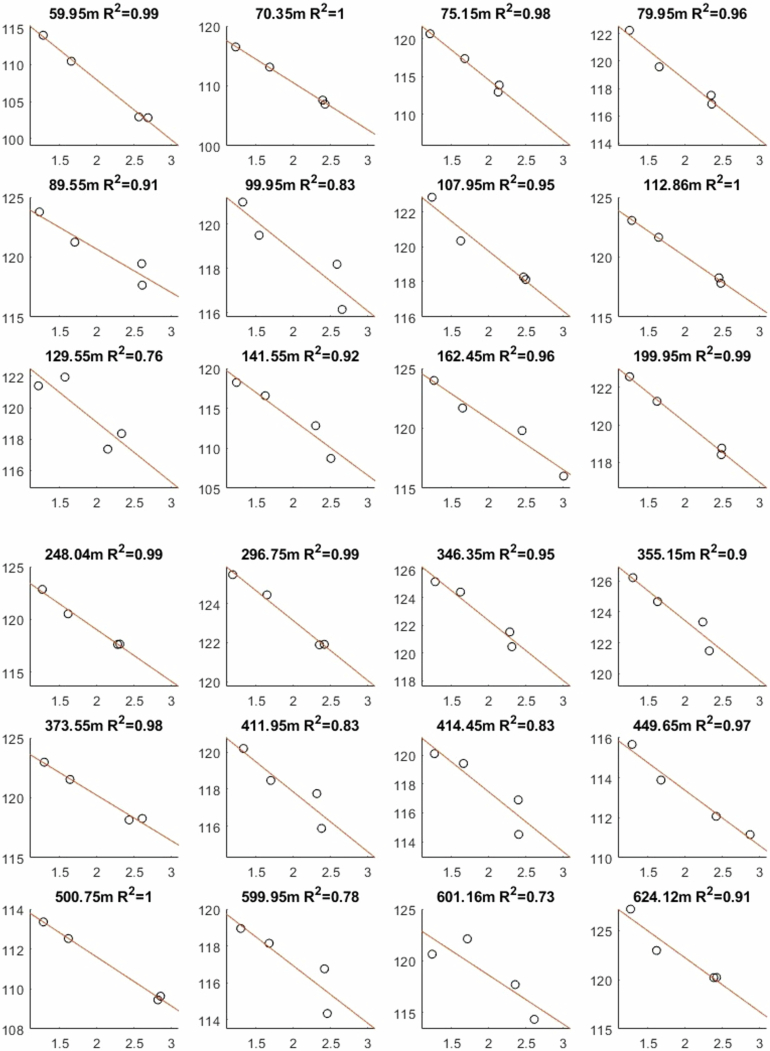
Plots used to estimate the cut-bubble correction for TAC at different depths. The y-axis is the measured air content (mL kg^−1^), while the x-axis is S/V (cm^−1^). See methods for explanation of how these plots were used.

TAC was measured using discrete samples of approximately 60 g of ice using a wet-extraction method^38^. Data (not corrected for cut bubbles) are shown in Extended Data Fig. 3. At various depths we used elongated cuboid samples of different dimensions to estimate the cut-bubble correction^48^; plotting measured TAC against the ratio of sample surface area to volume (*S*/*V*); the intercept is the corrected TAC for the given depth, whereas the gradient allows for correction of other samples with known *S*/*V* (Extended Data Fig. 6). Between 100 and 200 m (approximately 0.45–2 ka), the cut-bubble correction (excluding one outlier) is 6 ± 1.1% (mean, s.e.m.) for typical 60 g samples with dimensions 28.5 × 28.5 × 90 mm^3^. The data for ice of different sizes are too scattered at most depths to derive a robust relationship between the cut-bubble correction and depth (Extended Data Fig. 6). If we assume that the LIG and Holocene ice had similar numbers and shapes of bubbles at the lock-in depth, which is estimated at 48 m in both the Holocene and the LIG^35^, then we expect the cut-bubble correction to reduce as bubbles get smaller with depth (*D*), so that fewer of them intersect the edge of the sample, as *D*^−1/3^. This then implies that, to compare data from the LIG (about 600 m) with the late Holocene (100–200 m), we need to adjust LIG data by −2.8 ± 0.7%. We apply this adjustment to LIG data in the calculations of elevation that follow, but we refrain from correcting individual TAC values at this stage to avoid introducing assumptions about bubble number and shape for intermediate depths, including the last glacial maximum.

### Estimating how much isotopic change is due to elevation change

The pressure at the time and depth of close-off (closely related to atmospheric pressure) is given by:

2 $${P}_{{\rm{c}}}=\left(\frac{{\rm{TAC}}}{{V}_{{\rm{c}}}}\right)\times \left(\frac{{T}_{{\rm{c}}}}{{T}_{{\rm{S}}}}\right)\times {P}_{{\rm{S}}}$$

where TAC is the total air content after correction for cut bubbles, *V*_C_ and *T*_C_ are the pore volume per unit mass and temperature at close-off, and *T*_S_ and *P*_S_ normalize the data to standard temperature and pressure. *T*_c_ is calculated using the difference in δ^18^O between the LIG and early Holocene, and an isotopic lapse rate of 0.8 ± 0.2‰ per 100 m, which was estimated^38^ on the basis of earlier data and model studies^49,50^. Early work^51^ suggested that *V*_c_ increases with site temperature, at least spatially, with a relationship derived for warmer sites such as Skytrain of *V*_c_ = 4.5 × 10^−^^4^ × *T* + 0.02, where *V*_c_ is in ml kg^−1^ and *T* is temperature (K). However, it is not clear if this applies temporally. Site elevation for Antarctica can be estimated as −7588 × ln(*P*_c_/989.1) (ref. ^52^), so that the difference in elevation between two time periods (1 and 2) is

3 $${\Delta }_{{\rm{elev}}}=-\,\mathrm{7,588}\times {\rm{ln}}\left(\frac{{P}_{{\rm{c1}}}}{{P}_{{\rm{c2}}}}\right)$$

We then use TAC and δ^18^O in two sections in which TAC is relatively constant with depth (126–120 ka and 2–0.45 ka) along with the LIG cut-bubble adjustment described above and the δ^18^O/*T* gradient to estimate the elevation change using equations (2) and (3). We used a Monte Carlo calculation to propagate the uncertainties in the values of TAC at the two time periods, the cut-bubble adjustment and the δ^18^O/*T* gradient for two different cases, one in which *V*_c_ changes with *T* according to the spatial gradient and one in which it is invariant with temperature.

The small change in elevation between the LIG and Holocene is used in the text to estimate how much of the observed LIG–Holocene change in δ^18^O results from a lapse rate change in temperature. We used the same isotopic lapse rate as above.

### Water isotope modelling

Estimates of the expected change in water isotopes across Antarctica owing to the atmospheric circulation changes associated with a reduced or collapsed WAIS are taken from a paper^32^ that used the high-resolution Weather Research and Forecasting model with the addition of water isotope physics. For its main case, an Antarctic Ice Sheet (AIS) orography was used^39^ in which WAIS experienced total collapse. The Ronne and Ross ice shelf elevations were reduced to sea level although they remained ice-covered. The change in δ^18^O at SIR centred on 4.5‰, making it a particularly sensitive site to WAIS loss. As SIR in the provided orography was reduced in elevation by about 50 m, the elevation-corrected change in δ^18^O can be estimated for this model run as 4‰. A related modelling study (but with a lower resolution model and without isotopes) found that the atmospheric circulation response and associated surface temperature (and, by inference, water isotope) signal is linear as a function of elevation change^53^. This supports our inference that the ice loss for the real WAIS might have been only about half as much as for the ‘full collapse’ scenario. Finally, the high-resolution simulations^32^ indicate a reduction in deuterium excess (dxs = δD − 8δ^18^O) at SIR of about 1‰ with full collapse. Deuterium excess at SIR is 1.2‰ lower in the LIG (126–120 ka) than in the late Holocene (2–0 ka), using only our well-calibrated discrete samples, further supporting the interpretation that the SIR data do indicate some WAIS loss.

### Estimating ice shelf extent

To estimate the extent of the Ronne Ice Shelf, we followed the same procedure as in a recent study of the Holocene at SIR^38^. A thorough study of the concentration of sea salt ions with distance from the ice shelf edge on the Ronne Ice Shelf^37^ allows us (after converting their chloride values to the equivalent ssNa concentration using sea salt ratios) to derive a strong linear relationship of ln[ssNa] versus distance from the ice shelf edge (Fig. 2). This relationship is represented by the equation, [ssNa] = 460 × exp(−0.0029*x*), where *x* is distance from the edge of the ice shelf in kilometres. The present-day ssNa concentration of SIR falls very close to the best fit line. We then plot (Fig. 2) the concentrations for different times in the LIG to estimate the changing distance from the ice edge and therefore changing extents of the ice shelf.

Although distance from the source is a first-order control on sea salt, we acknowledge that sea ice extent, atmospheric lifetime and wind strength/direction play second-order roles and could alter the slope of the best fit line we show in Fig. 2c. This would somewhat alter the estimates of ice shelf extent we give, but such effects cannot plausibly negate the factor 10 increase in sea salt we would have expected if the ice shelf had disappeared.

In making these estimates, we assume that any change in elevation has a minimal effect on Na concentration compared to the effect of source distance. This is supported by previous work^54^ from shallow ice cores, which found no change in Na concentration across a 900-m gradient in elevation around Berkner Island. At SIR, sea salt did not significantly change during an early Holocene isotopic event which was interpreted as being a 400 m elevation change^38^.

We note that ice sheet model simulations in which ice from the Amundsen Sea sector of Antarctica is removed could place open water closer to SIR in that direction (for example, Fig. 4). However the existence of the Ellsworth Mountains and a remnant high elevation ice sheet around it would restrict transport from that direction. Additionally, atmospheric models indicate that the removal of WAIS ice tends to strengthen surface winds on the Ronne Ice Shelf from the Weddell Sea direction^32^. If some salt does reach SIR from the Amundsen Sea direction in the LIG this would further lower the contribution from the Weddell Sea, requiring an even more distant ice shelf edge than we have estimated.

### Ice sheet modelling

To assess whether it is possible to retain a Ronne Ice Shelf of a comparable extent to present throughout the LIG, while also allowing for the significant loss of WAIS as suggested by genomic data, we explore a previously published ensemble of LIG simulations^9^. Those experiments used the parallel ice sheet model (PISM)^55^ to transiently simulate the evolution of the AIS through the penultimate glacial maximum and into the LIG^28^. Taking environmental boundary conditions from transient CCSM3 ocean–atmosphere simulations for termination 1 as well as termination 2, the PISM experiments explored a range of glaciological and solid-earth parameters to find a combination with which ice sheet advance to last glacial maximum extent and retreat to present-day extent, could be captured. These parameters were then used to investigate how much more ice loss may have occurred during the LIG. It was concluded^9^ that the AIS could have sustained approximately 4 m sea-level-equivalent ice loss (compared to the modelled present-day extent using the same parameters), reaching a maximum at around 126 ka. Yet the LIG ice sheet configuration produced in that experiment was one of partial, not complete, WAIS collapse. Subsequent research shows that open seaways may have existed during the LIG that connected the Weddell, Amundsen and Ross Seas, necessitating more complete WAIS collapse. Here we present a scenario in which those open seaways are captured, while also allowing persistent ice shelves in the Weddell and Ross embayments. However, this scenario is based on a parameterization that uses an unrealistically viscous mantle (10^21^ Pa s^−1^). This parameterization does not reflect the much weaker mantle characteristics of WAIS that geophysical studies have shown, nor does it allow for a last glacial maximum-to-present simulation that fits well with observations. Because of these shortcomings, we emphasize that the reconstruction presented in Fig. 4 is not empirically validated. Its value, however, is in its internal glaciological consistency (the physics governing ice flow are robustly captured) and in its ability to demonstrate that such a configuration is physically plausible. No doubt there may be other parameterizations in which a similar ice sheet/shelf pattern could be reproduced while also fitting more closely to the present-day. Full details of model parameterization and simulation methodology are presented in previous papers^9,28^.

## Online content

Any methods, additional references, Nature Portfolio reporting summaries, source data, extended data, supplementary information, acknowledgements, peer review information; details of author contributions and competing interests; and statements of data and code availability are available at 10.1038/s41586-024-08394-w.

## Figures and Tables

**Extended Data Table 1:**
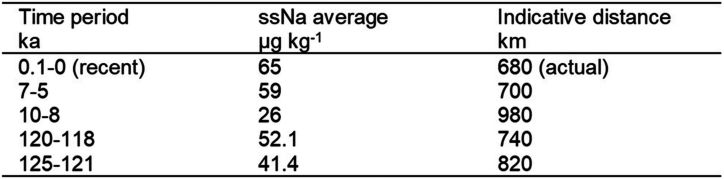
Concentrations of ssNa at different times at SIR

Indicative distance is the distance between SIR and the Ronne Ice Shelf edge calculated based on the best fit line of the spatial data^37^.

**Extended Data Table 2:**
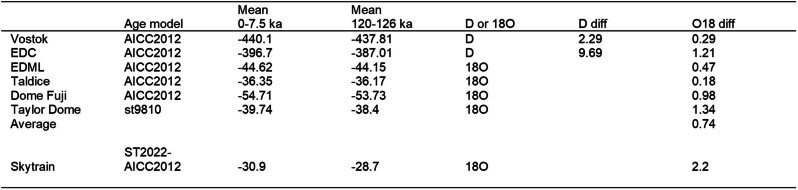
Differences in measured water isotope ratios between the LIG and the Holocene around Antarctica

Differences in δD are converted to equivalent differences in δ^18^O using a ratio of 8:1. Original isotopic data for Vostok^62^, EDC^6^, EDML^44^, Taldice^63^, Dome Fuji^64^ and Taylor Dome^65^.

## Data Availability

The water isotope, Na and TAC data presented in this paper are archived in the Pangaea database^56–59^. The map in Fig. 1 was generated using QGIS with the Quantarctica mapping environment, under a Creative Commons licence CC BY 4.0. The rock outcrop data in the Quantarctica environment, and shown in Fig. 1 are from ref. ^60^, European Geosciences Union, under a Creative Commons licence CC BY 3.0. The model output maps in Fig. 4 and Extended Data Fig. 4 were plotted using Generic Mapping Tools v.6.
