# Author Correction: The Ronne Ice Shelf survived the last interglacial

**DOI:** 10.1038/s41586-025-08806-5

**Published:** 2025-02-24

**Authors:** Eric W. Wolff, Robert Mulvaney, Mackenzie M. Grieman, Helene M. Hoffmann, Jack Humby, Christoph Nehrbass-Ahles, Rachael H. Rhodes, Isobel F. Rowell, Louise C. Sime, Hubertus Fischer, Thomas F. Stocker, Amaelle Landais, Frédéric Parrenin, Eric J. Steig, Marina Dütsch, Nicholas R. Golledge

**Affiliations:** 1 Department of Earth Sciences, University of Cambridge, Cambridge, UK; 2 British Antarctic Survey, Cambridge, UK; 3 Geo- and Environmental Center, University of Tübingen, Tübingen, Germany; 4 National Physical Laboratory, Teddington, UK; 5 Climate and Environmental Physics, Physics Institute, and Oeschger Centre for Climate Change, University of Bern, Bern, Switzerland; 6 Laboratoire des Sciences du Climat et de l’Environnement, LSCE/IPSL, CEA-CNRS-UVSQ, Université Paris-Saclay, Gif-sur-Yvette, France; 7 Université Grenoble Alpes, CNRS, INRAE, IRD, Grenoble INP, IGE, Grenoble, France; 8 Department of Earth and Space Sciences and Department of Atmospheric Sciences, University of Washington, Seattle, WA USA; 9 Department of Meteorology and Geophysics, University of Vienna, Vienna, Austria; 10 Antarctic Research Centre, Victoria University of Wellington, Wellington, New Zealand

**Keywords:** Palaeoclimate, Cryospheric science

Correction to: *Nature* 10.1038/s41586-024-08394-w Published online 29 January 2025

In the version of the article initially published, the DOI in ref. 56 was incorrect and has now been amended to 10.1594/PANGAEA.973166 in the HTML and PDF versions of the article.

